# Acute Kidney Injury Associated With Isolated C3 Complement Deficiency Following Hemorrhagic Ovarian Cyst Rupture: A Report of a Rare Case

**DOI:** 10.7759/cureus.90874

**Published:** 2025-08-24

**Authors:** Sevil Uygun İlikhan, Canan Güneş, Sevgi Kılınç, Sinem Ülke, Selma Karaahmetoğlu

**Affiliations:** 1 Internal Medicine, Ankara Bilkent Şehir Hastanesi, Ankara, TUR

**Keywords:** acute kidney injury, c3 deficiency, case report, complement system, hemorrhagic ovarian cyst

## Abstract

The complement system plays a crucial role in innate immunity and inflammation regulation, with component C3 being central to its activation. Isolated C3 deficiency is rare and may occur secondary to immune, infectious, or inflammatory conditions.

We present a 23-year-old woman admitted with nausea, vomiting, and right lower abdominal pain. Laboratory tests revealed acute kidney injury (creatinine: 1.71 mg/dL; urea: 64 mg/dL), and imaging identified a hemorrhagic ovarian cyst rupture with free peritoneal fluid. Complement testing revealed low serum C3 levels with normal C4 and negative autoimmune and infectious markers. The patient was treated conservatively with hydration, and renal function returned to normal within days.

This case highlights a rare association between hemorrhagic ovarian cyst rupture and acute kidney injury in the context of isolated C3 deficiency. Although no underlying systemic disease was detected, the transient complement activation may reflect an acute inflammatory response. Isolated complement abnormalities should be interpreted with clinical context and followed closely.

## Introduction

The complement system is a major component of innate immunity and plays a key role in inflammation and host defense. Complement component 3 (C3), primarily synthesized in the liver, serves as the central convergence point of all three complement activation pathways. Decreased serum C3 levels may result from congenital deficiency or secondary consumption due to autoimmune, infectious, or inflammatory diseases such as systemic lupus erythematosus (SLE), glomerulonephritis, post-streptococcal glomerulonephritis (APSGN), and infective endocarditis. In this report, we present a unique case of acute kidney injury (AKI) in the setting of isolated C3 deficiency and a ruptured hemorrhagic ovarian cyst. To our knowledge, the co-occurrence of these conditions has not been previously described in the literature. AKI is a clinically important condition even in young and otherwise healthy individuals. Its occurrence in association with isolated C3 deficiency and hemorrhagic ovarian cyst rupture highlights the need for careful evaluation in such unusual presentations.

## Case presentation

A 23-year-old woman presented to the emergency department with nausea, vomiting, and right-sided lower abdominal pain for the past two days. Her medical history was unremarkable. She initially visited another facility, where elevated serum creatinine was noted, and she was referred to our hospital for further evaluation.

On admission, she was alert and hemodynamically stable. Physical examination revealed localized tenderness in the right lower quadrant, particularly in the right inguinal region, without rebound or guarding. Laboratory analysis demonstrated leukocytosis (15.22×10⁹/L), mild anemia (Hb: 11.6 g/dL), and thrombocytopenia (126×10⁹/L), suggesting systemic inflammation. Serum creatinine was elevated at 1.71 mg/dL. Urinalysis revealed pyuria (66 leukocytes) and microscopic hematuria (20 erythrocytes), though urine and blood cultures were sterile (Table 1).

**Table 1:** Laboratory test results

Parameter	Patient's results	Reference range
Leukocytes	15.22×10⁹/L	3.6-10.5×10⁹/L
Hemoglobin	11.6 g/dL	11.8-15.8 g/dL
Platelets	126×10⁹/L	160-400×10⁹/L
Creatinine	1.71 mg/dL	0.5-1.1 mg/dL
Complement 3 (C3)	0.8 g/L	0.9-1.8 g/L
Complement 4 (C4)	0.2 g/L	0.1-0.4 g/L
Urinalysis: leukocytes	66/HPF	0-3/HPF
Urinalysis: erythrocytes	20/HPF	0-5/HPF

The urine microscopy showed red blood cells and leukocytes, but no erythrocyte or leukocyte casts were observed. This finding was not suggestive of glomerular pathology and supported a pre-renal etiology.

**Figure 1:**
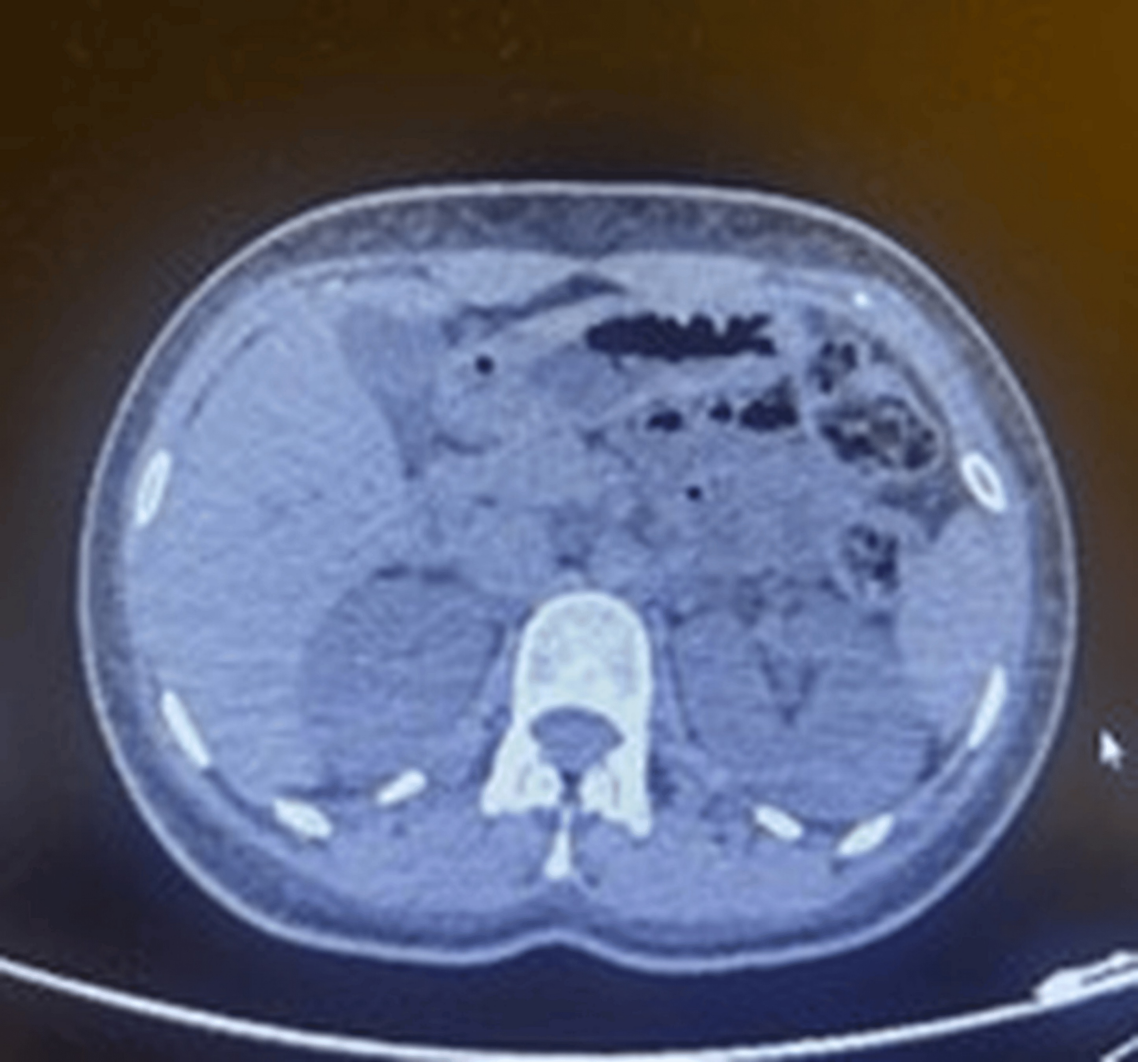
Abdominopelvic computed tomography scan showing free peritoneal fluid consistent with hemorrhagic ovarian cyst rupture The computed tomography image was acquired with informed consent obtained from the patient.

Abdominopelvic computed tomography revealed free fluid in the Douglas pouch and paraovarian spaces, along with a right ovarian hemorrhagic follicular cyst (Figure 1). Pelvic ultrasonography confirmed 4-5 cm of free fluid without signs of active bleeding. Conservative management was initiated with intravenous hydration only. No antibiotics, dialysis, or immunosuppressive therapy was administered. Renal function normalized rapidly with supportive care.

**Figure 2:**
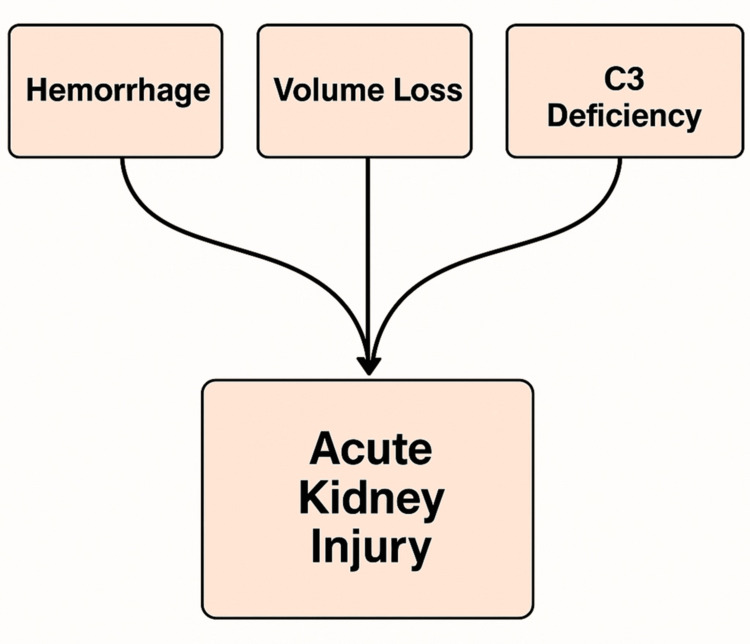
Pathophysiology of acute kidney injury This figure was created by the authors for illustrative purposes.

Complement studies revealed decreased C3 with normal C4. Autoimmune screening and viral serologies were negative. No renal biopsy was performed in this case. According to the Kidney Disease: Improving Global Outcomes (KDIGO) criteria, the patient had stage 2 AKI (serum creatinine increased 2-2.9 times baseline). Renal function returned to normal within days, and she was discharged in good condition (Figure 2).

## Discussion

AKI is defined as a sudden decline in glomerular filtration rate (GFR), leading to the accumulation of nitrogenous waste products such as urea and creatinine. In contrast to chronic kidney disease, where GFR declines progressively over months or years, AKI develops over hours to days. It can present with oliguria (<400 mL/day), anuria (<100 mL/day), or even normal urine output (non-oliguric AKI) [1]. The underlying mechanisms may be pre-renal, intrinsic renal, or post-renal.

In our patient, the rapid onset of renal dysfunction without structural abnormalities on imaging and in the absence of urinary casts suggests a pre-renal etiology, most likely secondary to intraperitoneal hemorrhage and subsequent volume depletion. Urinalysis demonstrated microscopic hematuria and pyuria, but importantly, no erythrocyte or leukocyte casts were observed, making intrinsic glomerular pathology unlikely. No renal biopsy was performed, further supporting that the episode represented a transient, pre-renal process rather than intrinsic renal injury. Hemorrhagic ovarian cyst rupture is a known cause of acute abdomen and can occasionally lead to hemodynamic instability due to significant blood loss [2]. In most cases, such cysts resolve spontaneously, and surgery is reserved for ongoing bleeding or hemodynamic compromise [3].

Complement component C3 plays a central role in innate immunity, particularly in opsonization and immune complex clearance. Decreased serum C3 levels may reflect inherited deficiency or increased consumption due to immune activation. In the absence of infection or autoimmune disease, as in this patient, low C3 levels most likely reflect transient complement activation triggered by acute inflammation and hemorrhage [4,5].

C3 glomerulopathies are characterized by dominant C3 deposition in the glomeruli and are often associated with persistent low C3 levels, proteinuria, hematuria, and progressive renal impairment [6]. However, our patient had no proteinuria, no urinary casts, and no biopsy evidence suggesting glomerular disease, and her C3 level was the only abnormal immunological finding.

We hypothesize that the isolated C3 deficiency in this case represents a temporary state of complement consumption associated with hemorrhage and tissue injury, rather than an underlying immunodeficiency or primary glomerular disorder. The rapid recovery of renal function and normalization of clinical parameters support this interpretation. Nevertheless, patients with isolated complement abnormalities should be monitored longitudinally to detect any potential development of immune-mediated disease.

## Conclusions

This case report describes a rare instance of AKI associated with isolated complement C3 deficiency following the rupture of a hemorrhagic ovarian cyst. The complement system, particularly component C3, plays a pivotal role in innate immunity and the regulation of inflammatory responses. In this case, the isolated reduction in serum C3 levels, in the absence of infectious or autoimmune etiologies, likely reflects transient complement consumption triggered by acute inflammation and hypovolemia resulting from hemorrhage.

From a clinical perspective, the rapid restoration of renal function supports a transient, pre-renal cause of AKI rather than intrinsic renal pathology. Moreover, the normalization of complement levels during follow-up argues against a chronic or inherited immunodeficiency. Nonetheless, the systemic response observed in this otherwise common gynecologic condition underscores the need to consider complement evaluation in cases of unexplained AKI.

This case illustrates that even an uncomplicated ovarian cyst rupture can occasionally result in significant systemic consequences, including renal dysfunction, particularly in susceptible individuals. Therefore, clinicians should maintain awareness of the potential immunologic ramifications in similar clinical presentations. Ultimately, isolated complement abnormalities, such as transient C3 deficiency, must be interpreted within the appropriate clinical context and monitored longitudinally to identify any emerging immune-mediated disorders.
